# The genome sequence of the meadow plant bug, *Leptopterna dolabrata *(Linnaeus, 1758)

**DOI:** 10.12688/wellcomeopenres.21005.1

**Published:** 2024-03-04

**Authors:** Liam M. Crowley, Laurence Livermore, University of Oxford and Wytham Woods Genome Acquisition Lab, Darwin Tree of Life Barcoding collective, Wellcome Sanger Institute Tree of Life Management, Samples and Laboratory team, Wellcome Sanger Institute Scientific Operations: Sequencing Operations, Wellcome Sanger Institute Tree of Life Core Informatics team, Tree of Life Core Informatics collective, Darwin Tree of Life Consortium

**Affiliations:** 1 University of Oxford, Oxford, England, UK; 2 Natural History Museum, London, England, UK

**Keywords:** Leptopterna dolabrata, meadow plant bug, genome sequence, chromosomal, Hemiptera

## Abstract

We present a genome assembly from an individual male *Leptopterna dolabrata* (the meadow plant bug; Arthropoda; Insecta; Hemiptera; Miridae). The genome sequence is 987.9 megabases in span. Most of the assembly is scaffolded into 17 chromosomal pseudomolecules, including the X sex chromosome. The mitochondrial genome has also been assembled and is 18.18 kilobases in length.

## Species taxonomy

Eukaryota; Opisthokonta; Metazoa; Eumetazoa; Bilateria; Protostomia; Ecdysozoa; Panarthropoda; Arthropoda; Mandibulata; Pancrustacea; Hexapoda; Insecta; Dicondylia; Pterygota; Neoptera; Paraneoptera; Hemiptera; Prosorrhyncha; Heteroptera; Euheteroptera; Neoheteroptera; Panheteroptera; Cimicomorpha; Cimicoidea; Miridae; Mirinae; Stenodemini; *Leptopterna*; *Leptopterna dolabrata* ( Linnaeus, 1758) (NCBI:txid881489).

## Background

The meadow plant bug, *Leptopterna dolabrata* ( Linnaeus, 1758), is a heteropteran bug of length 8.3–9.8 mm ( Scudder & Schwartz, 2001). Adults are elongate and pale yellow to red-orange (the colour darkens with age), with black longitudinal markings on the head, thorax, and abdomen. Its legs and antennae are covered with dark hairs. The hemelytra have similar colouration to the rest of the body, also with black longitudinal markings. It is a sexually dimorphic species where the males are always macropterous (long-winged) and the females mostly brachypterous (short-winged). In macropters, the hemelytral membrane is dark/black with venation matching the general body colour around the membrane cells. It looks similar to the closely-related and co-occurring *L. ferrugata* and can be identified by antennae: in the first antennal segment *L. dolabrata* has stiff black hairs with some longer and projecting outwards while *L. ferrugata* has more evenly distributed hairs of equal length that recline against the antennae; in *L. dolabrata* the length of the second antennal segment is greater than the combined length of the third and fourth ( Scudder & Schwartz, 2001). While less reliable for identification, the adults of *L. ferrugata* have pink to light brown wings and prefer dryer grassland habitats compared to *L. dolabrata*.

Originally described as *Cimex dolabratus* by Linnaeus in 1758 ( Linnaeus, 1758: 449), the genus *Leptopterna* was raised by Fieber in 1858 ( Fieber, 1858: 302) with *L. dolabrata* as the only species.

*Leptopterna dolabrata* is widespread across the UK, spanning the south coast of England to the Outer Hebrides ( NBN Atlas Partnership, 2023; Ryan, 2023). It occurs throughout Europe with its easterly range extending to Kazakhstan and West Siberia ( Kerzhner & Josifov, 1999:186–187). Further east it is replaced by *L. kerzhneri* Vinokurov, 1982 and *L. ruficornis* Vinokurov, 1882 ( Vinokurov, 2019). It is an introduced and established species in eastern and western North America ( Henry & Froeschner, 1988: 384; Scudder & Schwartz, 2001). *Leptopterna dolabrata* prefers moist conditions and can be found in meadows and grassy places.

*Leptopterna dolabrata* lays its eggs in the lower part of stems of its host grasses. The eggs overwinter and undergo obligatory diapause and, in the UK, start hatching in May ( Saulich & Musolin, 2020; Southwood & Leston, 1959: 312). Adults occur in June onwards and are most frequently seen in June and July ( NBN Atlas Partnership, 2023).

In the UK its food plants are a range of common grasses including *Phleum pratense* L., *Elymus repens* (L.), *Alopecurus pratensis* L., *Dactylis glomerata* L., and *Holcus lanatus* L. ( Southwood & Leston, 1959: 312). *Leptopterna dolabrata* has been associated with the transmission of silvertop disease of grasses, which in severe cases can destroy the entire inflorescence. Direct transfer of the most common causal biotic agent of silvertop, the fungus *Fusarium poae,* by *L. dolabrata* is suspected but remains to be demonstrated ( Nedelník *et al.*, 2015). It is an occasional pest of cereal crops, including corn (maize), rye and wheat ( Knowlton, 1947; Wheeler, 2001: 195, 200).

Southwood and Leston report the diploid (2 *n*) karyotype of *Leptopterna dolabrata* to be 34, comprising of 32 autosomes and two sex chromosomes (XY) ( Southwood & Leston, 1959: 313). The genome of the meadow plant bug, *Leptopterna dolabrata*, was sequenced as part of the Darwin Tree of Life Project, a collaborative effort to sequence all named eukaryotic species in the Atlantic Archipelago of Britain and Ireland.

## Genome sequence report

**Figure 1.**
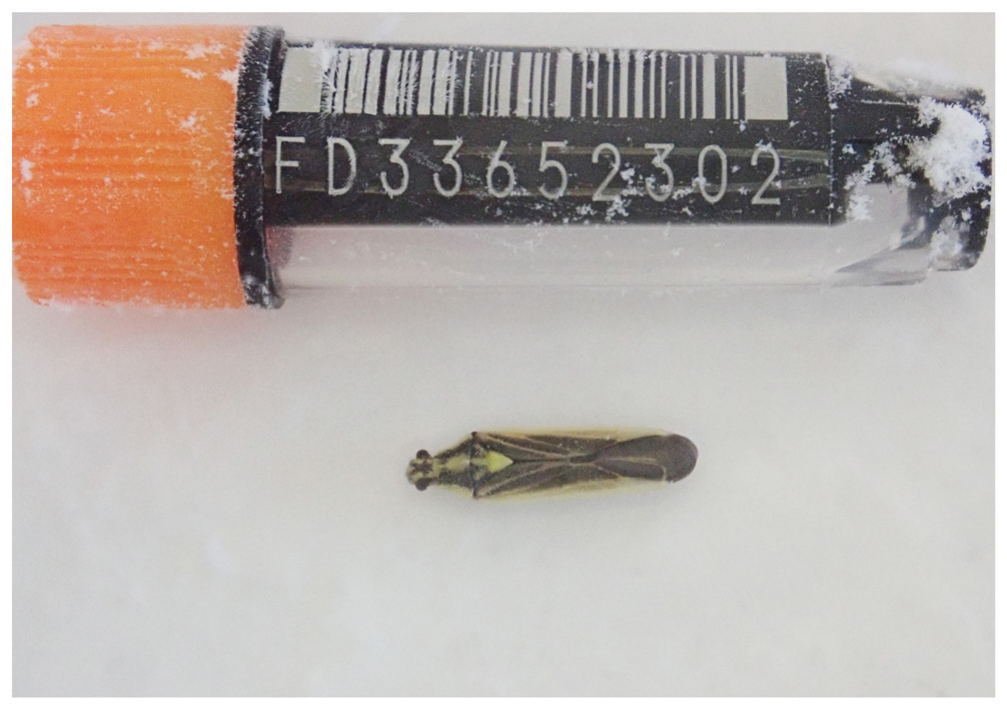
Photograph of the *Leptopterna dolabrata* (ihLepDola1) specimen used for genome sequencing.

The genome was sequenced from one male *Leptopterna dolabrata* ( Figure 1) collected from Oxfordshire, UK (51.77, –1.27). A total of 23-fold coverage in Pacific Biosciences single-molecule HiFi long reads was generated. Primary assembly contigs were scaffolded with chromosome conformation Hi-C data. Manual assembly curation corrected 104 missing joins or mis-joins and removed 12 haplotypic duplications, reducing the assembly length by 0.44% and the scaffold number by 7.42%, and decreasing the scaffold N50 by 1.27%.

**Figure 2.**
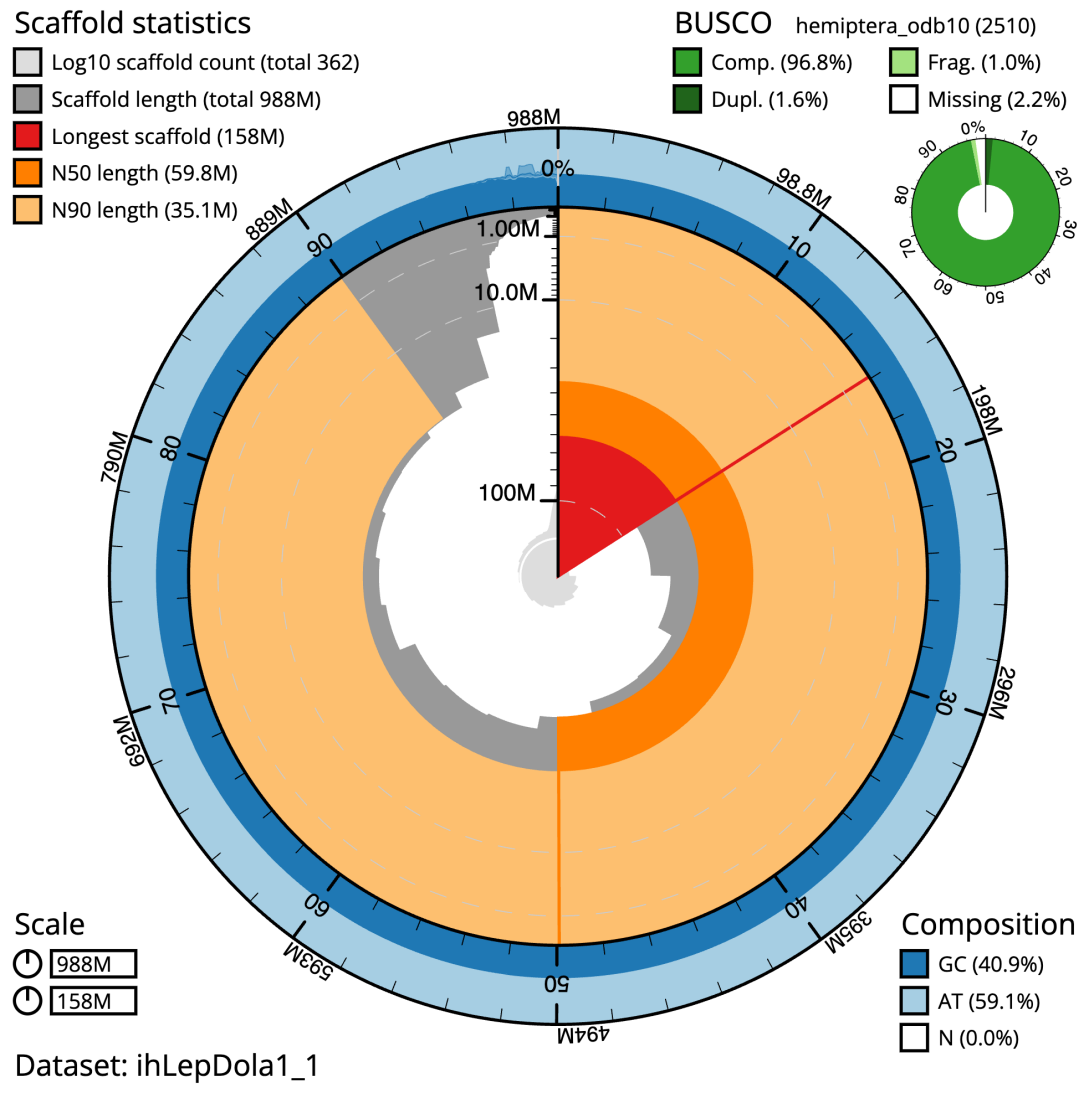
Genome assembly of *Leptopterna dolabrata*, ihLepDola1.1: metrics. The BlobToolKit Snailplot shows N50 metrics and BUSCO gene completeness. The main plot is divided into 1,000 size-ordered bins around the circumference with each bin representing 0.1% of the 987,919,866 bp assembly. The distribution of scaffold lengths is shown in dark grey with the plot radius scaled to the longest scaffold present in the assembly (157,922,925 bp, shown in red). Orange and pale-orange arcs show the N50 and N90 scaffold lengths (59,806,621 and 35,100,493 bp), respectively. The pale grey spiral shows the cumulative scaffold count on a log scale with white scale lines showing successive orders of magnitude. The blue and pale-blue area around the outside of the plot shows the distribution of GC, AT and N percentages in the same bins as the inner plot. A summary of complete, fragmented, duplicated and missing BUSCO genes in the hemiptera_odb10 set is shown in the top right. An interactive version of this figure is available at https://blobtoolkit.genomehubs.org/view/ihLepDola1_1/dataset/ihLepDola1_1/snail.

**Figure 3.**
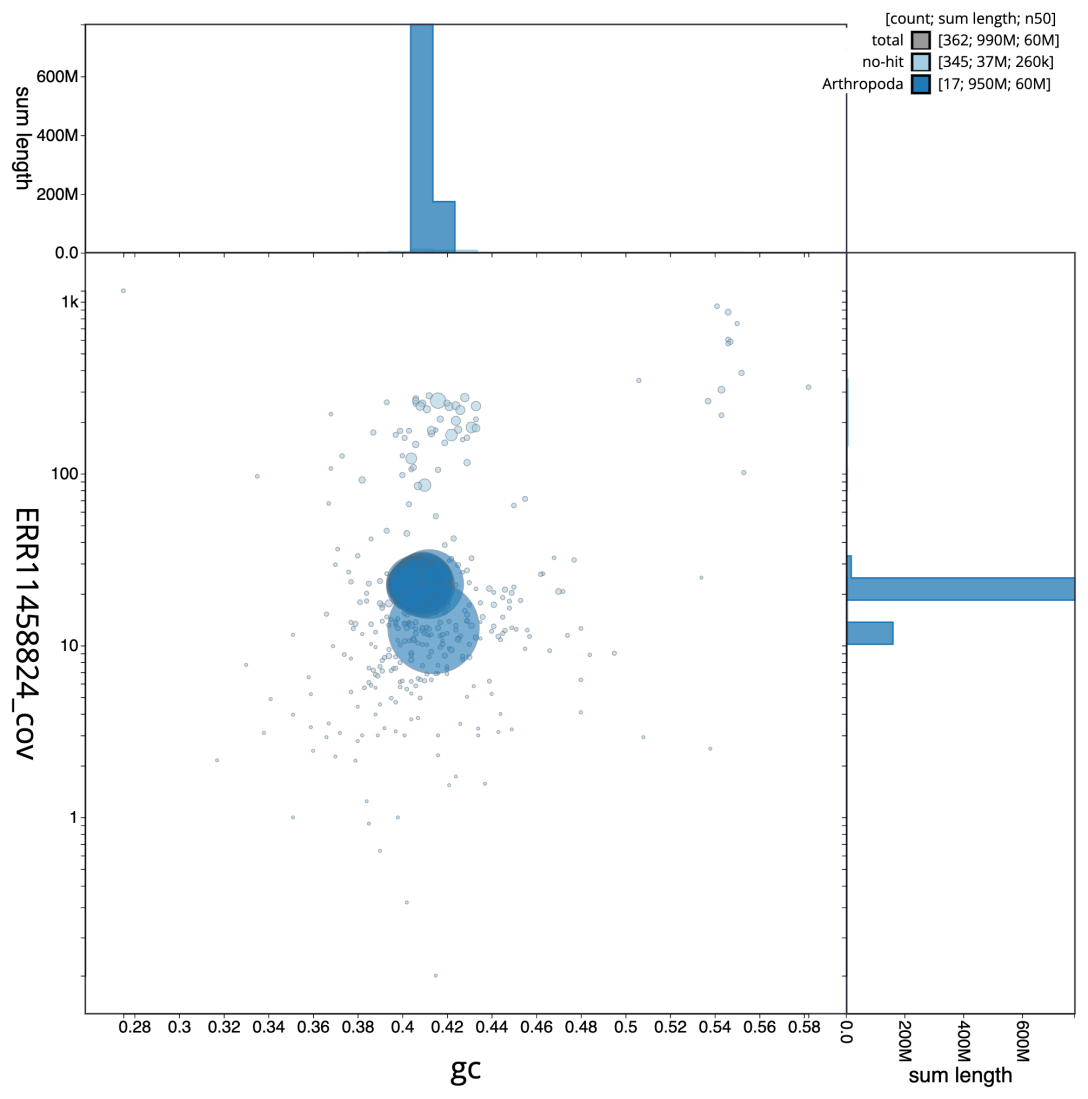
Genome assembly of *Leptopterna dolabrata*, ihLepDola1.1: BlobToolKit GC-coverage plot. Scaffolds are coloured by phylum. Circles are sized in proportion to scaffold length. Histograms show the distribution of scaffold length sum along each axis. An interactive version of this figure is available at https://blobtoolkit.genomehubs.org/view/ihLepDola1_1/dataset/ihLepDola1_1/blob.

**Figure 4.**
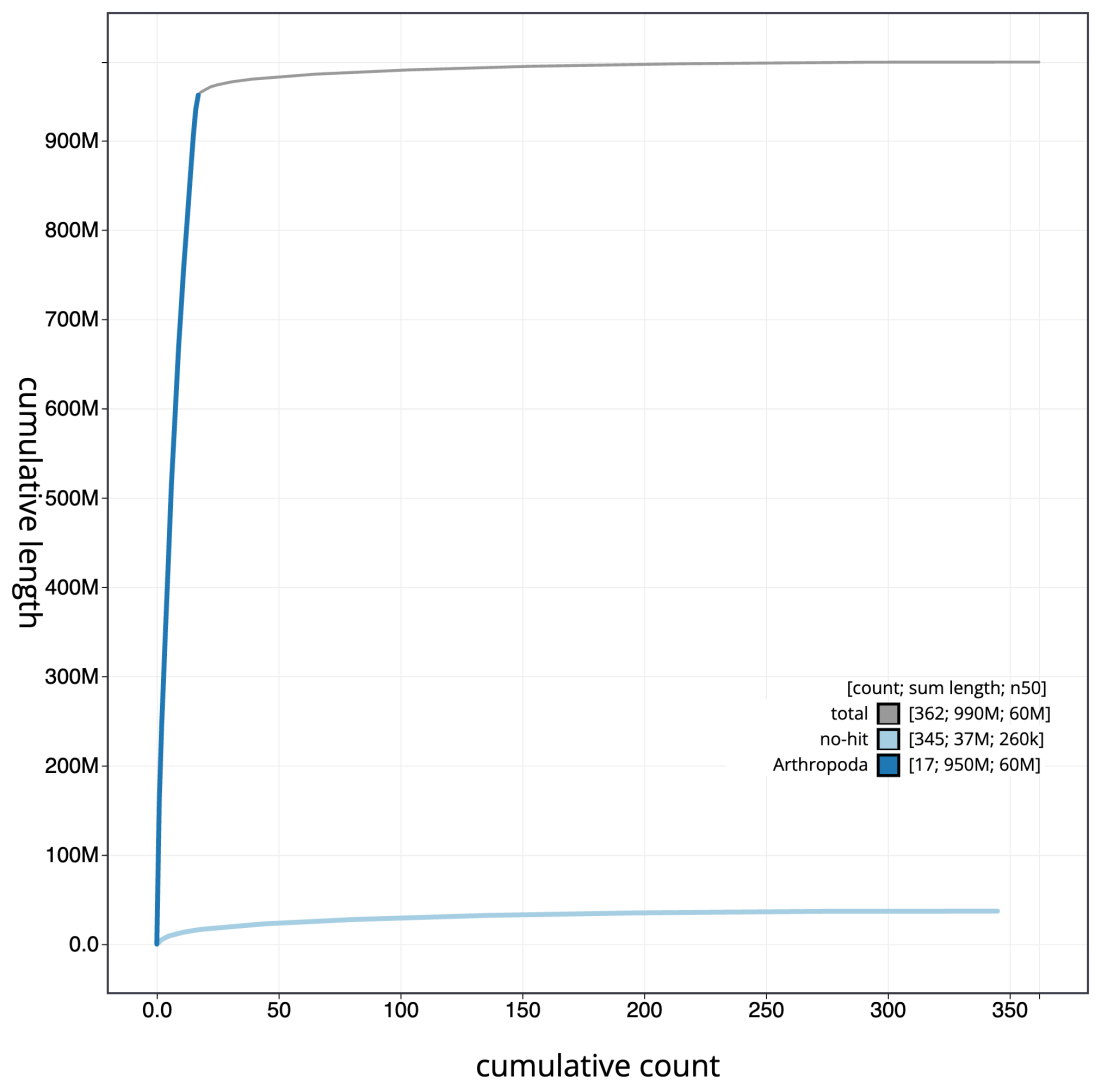
Genome assembly of *Leptopterna dolabrata*, ihLepDola1.1: BlobToolKit cumulative sequence plot. The grey line shows cumulative length for all scaffolds. Coloured lines show cumulative lengths of scaffolds assigned to each phylum using the buscogenes taxrule. An interactive version of this figure is available at https://blobtoolkit.genomehubs.org/view/ihLepDola1_1/dataset/ihLepDola1_1/cumulative.

**Figure 5.**
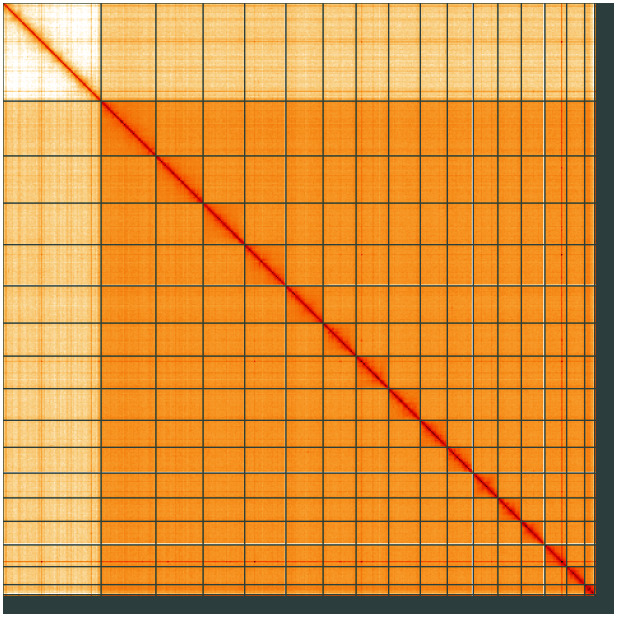
Genome assembly of *Leptopterna dolabrata*, ihLepDola1.1: Hi-C contact map of the ihLepDola1.1 assembly, visualised using HiGlass. Chromosomes are shown in order of size from left to right and top to bottom. An interactive version of this figure may be viewed at https://genome-note-higlass.tol.sanger.ac.uk/l/?d=BmvY09zZR966lCB0d13ZFA.

The final assembly has a total length of 987.9 Mb in 361 sequence scaffolds with a scaffold N50 of 59.8 Mb ( Table 1). The snailplot in Figure 2 provides a summary of the assembly statistics, while the distribution of assembly scaffolds on GC proportion and coverage is shown in Figure 3. The cumulative assembly plot in Figure 4 shows curves for subsets of scaffolds assigned to different phyla. Most (96.25%) of the assembly sequence was assigned to 17 chromosomal-level scaffolds, representing 16 autosomes and the X sex chromosome. Chromosome-scale scaffolds confirmed by the Hi-C data are named in order of size ( Figure 5; Table 2). The X chromosome was assigned based on read coverage statistics. This species appears to be an XO male. While not fully phased, the assembly deposited is of one haplotype. Contigs corresponding to the second haplotype have also been deposited. The mitochondrial genome was also assembled and can be found as a contig within the multifasta file of the genome submission.

**Table 1.** Genome data for *Leptopterna dolabrata*, ihLepDola1.1.

Project accession data		
Assembly identifier	ihLepDola1.1	
Species	*Leptopterna dolabrata*	
Specimen	ihLepDola1	
NCBI taxonomy ID	881489	
BioProject	PRJEB62413	
BioSample ID	SAMEA112226472	
Isolate information	ihLepDola1, male: whole organism (DNA and Hi-C sequencing)	
Assembly metrics *		*Benchmark*
Consensus quality (QV)	56.8	*≥ 50*
*k*-mer completeness	99.99%	*≥ 95%*
BUSCO **	C:96.8%[S:95.3%,D:1.6%],F:1.0%,M:2.2%,n:2,510	*C ≥ 95%*
Percentage of assembly mapped to chromosomes	96.25%	*≥ 95%*
Sex chromosomes	XO	*localised homologous pairs*
Organelles	Mitochondrial genome: 18.18 kb	*complete single alleles*
Raw data accessions		
PacificBiosciences SEQUEL II	ERR11458824	
Hi-C Illumina	ERR11468758	
Genome assembly		
Assembly accession	GCA_954871275.1	
*Accession of alternate haplotype*	GCA_954871245.1	
Span (Mb)	987.9	
Number of contigs	2,063	
Contig N50 length (Mb)	1.0	
Number of scaffolds	361	
Scaffold N50 length (Mb)	59.8	
Longest scaffold (Mb)	157.92	

* Assembly metric benchmarks are adapted from column VGP-2020 of “Table 1: Proposed standards and metrics for defining genome assembly quality” from ( Rhie *et al.*, 2021). ** BUSCO scores based on the hemiptera_odb10 BUSCO set using version 5.3.2. C = complete [S = single copy, D = duplicated], F = fragmented, M = missing, n = number of orthologues in comparison. A full set of BUSCO scores is available at https://blobtoolkit.genomehubs.org/view/ihLepDola1_1/dataset/ihLepDola1_1/busco.

**Table 2.** Chromosomal pseudomolecules in the genome assembly of *Leptopterna dolabrata*, ihLepDola1.

INSDC accession	Chromosome	Length (Mb)	GC%
OX940975.1	1	87.91	41.0
OX940976.1	2	75.81	41.0
OX940977.1	3	66.89	41.0
OX940978.1	4	66.15	41.0
OX940979.1	5	59.81	41.0
OX940980.1	6	53.21	40.5
OX940981.1	7	52.15	40.5
OX940982.1	8	51.16	40.5
OX940983.1	9	43.5	40.5
OX940984.1	10	41.47	40.5
OX940985.1	11	39.62	40.5
OX940986.1	12	37.86	40.5
OX940987.1	13	37.58	40.5
OX940988.1	14	35.1	40.5
OX940989.1	15	28.95	40.5
OX940990.1	16	15.86	41.5
OX940974.1	X	157.92	41.5
OX940991.1	MT	0.02	28.0

The estimated Quality Value (QV) of the final assembly is 56.8 with *k*-mer completeness of 99.99%, and the assembly has a BUSCO v5.3.2 completeness of 96.8% (single = 95.3%, duplicated = 1.6%), using the hemiptera_odb10 reference set ( *n* = 2,510).

Metadata for specimens, barcode results, spectra estimates, sequencing runs, contaminants and pre-curation assembly statistics are given at https://links.tol.sanger.ac.uk/species/881489.

## Methods

### Sample acquisition and nucleic acid extraction

A male *Leptopterna dolabrata* (specimen ID Ox002246, ToLID ihLepDola1) was collected from Trap Grounds, Oxfordshire, UK (latitude 51.77, longitude –1.27) on 2022-06-07 using a sweep net. The specimen was collected and identified by Liam Crowley (University of Oxford) and preserved on dry ice.

The workflow for high molecular weight (HMW) DNA extraction at the WSI includes a sequence of core procedures: sample preparation; sample homogenisation, DNA extraction, fragmentation, and clean-up. In sample preparation, the ihLepDola1 sample was weighed and dissected on dry ice ( Jay *et al.*, 2023). Tissue of the whole organism was homogenised using a PowerMasher II tissue disruptor, setting aside tissue for Hi-C sequencing ( Denton *et al.*, 2023a). HMW DNA was extracted in the WSI Scientific Operations core using the Automated MagAttract v2 protocol ( Oatley *et al.*, 2023). The DNA was sheared into an average fragment size of 12–20 kb in a Megaruptor 3 system with speed setting 31 ( Bates *et al.*, 2023). Sheared DNA was purified by solid-phase reversible immobilisation ( Strickland *et al.*, 2023): in brief, the method employs a 1.8X ratio of AMPure PB beads to sample to eliminate shorter fragments and concentrate the DNA. The concentration of the sheared and purified DNA was assessed using a Nanodrop spectrophotometer and Qubit Fluorometer and Qubit dsDNA High Sensitivity Assay kit. Fragment size distribution was evaluated by running the sample on the FemtoPulse system.

Protocols developed by the Tree of Life laboratory are publicly available on protocols.io ( Denton *et al.*, 2023b).

### Sequencing

Pacific Biosciences HiFi circular consensus DNA sequencing libraries were constructed according to the manufacturers’ instructions. DNA sequencing was performed by the Scientific Operations core at the WSI on a Pacific Biosciences SEQUEL II instrument. Hi-C data were also generated from remaining tissue of ihLepDola1 using the Arima2 kit and sequenced on the Illumina NovaSeq 6000 instrument.

### Genome assembly, curation and evaluation

Assembly was carried out with Hifiasm ( Cheng *et al.*, 2021) and haplotypic duplication was identified and removed with purge_dups ( Guan *et al.*, 2020). The assembly was then scaffolded with Hi-C data ( Rao *et al.*, 2014) using YaHS ( Zhou *et al.*, 2023). The assembly was checked for contamination and corrected as described previously ( Howe *et al.*, 2021). Manual curation was performed using HiGlass ( Kerpedjiev *et al.*, 2018) and PretextView ( Harry, 2022). The mitochondrial genome was assembled using MitoHiFi ( Uliano-Silva *et al.*, 2023), which runs MitoFinder ( Allio *et al.*, 2020) or MITOS ( Bernt *et al.*, 2013) and uses these annotations to select the final mitochondrial contig and to ensure the general quality of the sequence.

A Hi-C map for the final assembly was produced using bwa-mem2 ( Vasimuddin *et al.*, 2019) in the Cooler file format ( Abdennur & Mirny, 2020). To assess the assembly metrics, the *k*-mer completeness and QV consensus quality values were calculated in Merqury ( Rhie *et al.*, 2020). This work was done using Nextflow ( Di Tommaso *et al.*, 2017) DSL2 pipelines “sanger-tol/readmapping” ( Surana *et al.*, 2023a) and “sanger-tol/genomenote” ( Surana *et al.*, 2023b). The genome was analysed within the BlobToolKit environment ( Challis *et al.*, 2020) and BUSCO scores ( Manni *et al.*, 2021; Simão *et al.*, 2015) were calculated.

Table 3 contains a list of relevant software tool versions and sources.

**Table 3.** Software tools: versions and sources.

Software tool	Version	Source
BlobToolKit	4.2.1	https://github.com/blobtoolkit/blobtoolkit
BUSCO	5.3.2	https://gitlab.com/ezlab/busco
Hifiasm	0.16.1-r375	https://github.com/chhylp123/hifiasm
HiGlass	1.11.6	https://github.com/higlass/higlass
Merqury	MerquryFK	https://github.com/thegenemyers/MERQURY.FK
MitoHiFi	3	https://github.com/marcelauliano/MitoHiFi
PretextView	0.2	https://github.com/wtsi-hpag/PretextView
purge_dups	1.2.5	https://github.com/dfguan/purge_dups
sanger-tol/genomenote	v1.0	https://github.com/sanger-tol/genomenote
sanger-tol/readmapping	1.1.0	https://github.com/sanger-tol/readmapping/tree/1.1.0
YaHS	1.2a.2	https://github.com/c-zhou/yahs

### Wellcome Sanger Institute – Legal and Governance

The materials that have contributed to this genome note have been supplied by a Darwin Tree of Life Partner. The submission of materials by a Darwin Tree of Life Partner is subject to the **‘Darwin Tree of Life Project Sampling Code of Practice’**, which can be found in full on the Darwin Tree of Life website here. By agreeing with and signing up to the Sampling Code of Practice, the Darwin Tree of Life Partner agrees they will meet the legal and ethical requirements and standards set out within this document in respect of all samples acquired for, and supplied to, the Darwin Tree of Life Project. 

Further, the Wellcome Sanger Institute employs a process whereby due diligence is carried out proportionate to the nature of the materials themselves, and the circumstances under which they have been/are to be collected and provided for use. The purpose of this is to address and mitigate any potential legal and/or ethical implications of receipt and use of the materials as part of the research project, and to ensure that in doing so we align with best practice wherever possible. The overarching areas of consideration are:

•   Ethical review of provenance and sourcing of the material

•   Legality of collection, transfer and use (national and international)

Each transfer of samples is further undertaken according to a Research Collaboration Agreement or Material Transfer Agreement entered into by the Darwin Tree of Life Partner, Genome Research Limited (operating as the Wellcome Sanger Institute), and in some circumstances other Darwin Tree of Life collaborators.

## Data Availability

European Nucleotide Archive: *Leptopterna dolabrata* (meadow plant bug). Accession number PRJEB62413; https://identifiers.org/ena.embl/PRJEB62413 ( Wellcome Sanger Institute, 2023). The genome sequence is released openly for reuse. The *Leptopterna dolabrata* genome sequencing initiative is part of the Darwin Tree of Life (DToL) project. All raw sequence data and the assembly have been deposited in INSDC databases. The genome will be annotated using available RNA-Seq data and presented through the Ensembl pipeline at the European Bioinformatics Institute. Raw data and assembly accession identifiers are reported in Table 1.

## Author information

Members of the University of Oxford and Wytham Woods Genome Acquisition Lab are listed here: https://doi.org/10.5281/zenodo.7125292.

Members of the Darwin Tree of Life Barcoding collective are listed here: https://doi.org/10.5281/zenodo.4893703.

Members of the Wellcome Sanger Institute Tree of Life Management, Samples and Laboratory team are listed here: https://doi.org/10.5281/zenodo.10066175.

Members of Wellcome Sanger Institute Scientific Operations: Sequencing Operations are listed here: https://doi.org/10.5281/zenodo.10043364.

Members of the Wellcome Sanger Institute Tree of Life Core Informatics team are listed here: https://doi.org/10.5281/zenodo.10066637.

Members of the Tree of Life Core Informatics collective are listed here: https://doi.org/10.5281/zenodo.5013541.

Members of the Darwin Tree of Life Consortium are listed here: https://doi.org/10.5281/zenodo.4783558.
