# Supplementary material for: The genome sequence of the meadow plant bug, Leptopterna dolabrata (Linnaeus, 1758)
Source: Wellcome Open Res. 2024 Mar 4;9:128. [Version 1] doi: 10.12688/wellcomeopenres.21005.1 (PMC11480713; doi:10.12688/wellcomeopenres.21005.1)
Supplement: Peer review history

# Reviewer report: Reviewer response for version 1

**DOI:** 10.21956/wellcomeopenres.23240.r103340

**Published:** 2024-10-15

**Authors:** Lim Li

**Affiliations:** 1 Universiti Sains Malaysia, George Town, Malaysia

The article is well-written, the data is well-presented, and I recommend it for indexing. I just have a small suggestion: Can the author replace Figure 1? Take a proper photo of the insect using an adequate camera tool under a dissecting microscope and put an appropriate scale bar, or just remove Figure 1.

Are sufficient details of methods and materials provided to allow replication by others?

Yes

Is the rationale for creating the dataset(s) clearly described?

Yes

Are the datasets clearly presented in a useable and accessible format?

Yes

Are the protocols appropriate and is the work technically sound?

Yes

Reviewer Expertise:

Molecular Biology, Entomology, Plant Biology, Plant Pathology

I confirm that I have read this submission and believe that I have an appropriate level of expertise to confirm that it is of an acceptable scientific standard.

# Reviewer report: Reviewer response for version 1

**DOI:** 10.21956/wellcomeopenres.23240.r94516

**Published:** 2024-09-06

**Authors:** Qianquan Chen

**Affiliations:** 1 Guizhou Normal University, Guiyang, Guizhou, China

As part of the Darwin Tree of Life Project, a collaborative effort to sequence all named eukaryotic species in the Atlantic Archipelago of Britain and Ireland, authors decoded the genome of meadow plant bug. They described methods in detail so that the readers could judge the reliability of the results. The genome sequence can contribute to the study of the meadow plant bug.

Are sufficient details of methods and materials provided to allow replication by others?

Yes

Is the rationale for creating the dataset(s) clearly described?

Yes

Are the datasets clearly presented in a useable and accessible format?

Yes

Are the protocols appropriate and is the work technically sound?

Yes

Reviewer Expertise:

Ecological Genomics

I confirm that I have read this submission and believe that I have an appropriate level of expertise to confirm that it is of an acceptable scientific standard.
